# Secure distributed genome analysis for GWAS and sequence comparison computation

**DOI:** 10.1186/1472-6947-15-S5-S4

**Published:** 2015-12-21

**Authors:** Yihua Zhang, Marina Blanton, Ghada Almashaqbeh

**Affiliations:** 1 Department of Computer Science and Engineering, University of Notre Dame, Notre Dame, USA; 2 Department of Computer Science, Columbia University, New York, USA

**Keywords:** secure genome analysis, iDASH competition, Hamming distance, oblivious sorting, oblivious merge, GWAS computation, secret sharing

## Abstract

**Background:**

The rapid increase in the availability and volume of genomic data makes significant advances in biomedical research possible, but sharing of genomic data poses challenges due to the highly sensitive nature of such data. To address the challenges, a competition for secure distributed processing of genomic data was organized by the iDASH research center.

**Methods:**

In this work we propose techniques for securing computation with real-life genomic data for minor allele frequency and chi-squared statistics computation, as well as distance computation between two genomic sequences, as specified by the iDASH competition tasks. We put forward novel optimizations, including a generalization of a version of mergesort, which might be of independent interest.

**Results:**

We provide implementation results of our techniques based on secret sharing that demonstrate practicality of the suggested protocols and also report on performance improvements due to our optimization techniques.

**Conclusions:**

This work describes our techniques, findings, and experimental results developed and obtained as part of iDASH 2015 research competition to secure real-life genomic computations and shows feasibility of securely computing with genomic data in practice.

## Background

### Introduction

The iDASH (Integrating Data for Analysis, Anonymization and SHaring) research center at the University of California, San Diego hosts an annual competition, which in 2015 was dedicated to secure genome analysis. The two challenges corresponded to secure non-interactive analysis of genomic data based on homomorphic encryption and secure interactive analysis using secure multi-party computation techniques. We focus on the second challenge and report our design and implementation of the competitions tasks, which consisted of distributed GWAS (Genome-Wide Association Study) computation and secure sequence comparisons in the form of the Hamming distance or edit distance. Here, GWAS is a study of common genetic variants in different individuals using case and control groups to determine if any variant is associated with a specific trait or genetic conditions.

We utilize secure multi-party computation (SMC) techniques based on secret sharing with lightweight computational footprints. This requires that all computation carried out jointly by the parties (i.e., computation that cannot be performed locally by data owners) is data-oblivious, which means that all instructions and accessed memory locations must be independent of the data. While this does not pose a challenge for some simpler computational tasks, meeting this objective often involves using non-trivial techniques for more complex functionalities. In particular, computing both Hamming and edit distances of two genome sequences involves a form of aligning the input sequences which is not straightforward to achieve in secure setting. A logical tool to resort to is to utilize secure set intersection for computing chromosome positions that appear in both input sequences, for which both two-party and multi-party implementations are known. Due to the specifics of our setting, we rely on the ideas from [1] for computing the set of positions common to both sequences, which in turn utilize oblivious sorting. The fastest oblivious sorting mechanism available to us at the time of competition preparation was sorting based on Batcher's mergesort [2], which works only on input sets, the size of which is a power of 2. This posed a problem because padding an input set of a large size to have the size equal to a power of 2 often can result in a significant performance slowdown which we wanted to overcome. Thus, the most challenging component of distance computation was generalizing the mergesort (more precisely, the merging step as described later) component of the computation to work with inputs of arbitrary sizes, which might be of independent interest. This and other optimizations and design decisions constitute the main contribution of this work. We also report on performance of our algorithms on real genome data.

### Tasks of the challenge

The challenge for secure multi-party computation based genomic data analysis had two tasks:

1 The first task was to develop secure distributed protocols for GWAS computations. The input consists of the genotypes of two groups of individuals (one case and one control group) over a number of Single Nucleotide Polymorphisms (SNP) with each of them being a DNA sequence variation, where a single nucleotide (A, T, C, or G) in the genome differs between individuals. The input is horizontally partitioned among two sites (e.g., two institutions, medical facilities, etc.), where each site cannot reveal its input to other parties. The task consists of securely computing minor allele frequencies (MAF) and chi-squared statistics for each of the SNPs in the case and control groups distributed across the two input parties. We provide the details of the computation below.

2 The second task was to develop secure distributed protocols for genomic sequence comparisons. The input consists of two genomic datasets, one from each individual, which are organized as the genotypes over many SNPs across the whole human genome. Each genomic dataset belongs to a different entity, and the data owner cannot reveal any information about its data to other parties. The task consists of securely computing either the Hamming distance or edit distance between the two genomic datasets, and we concentrate on Hamming distance computation. The computation involved in the Hamming distance computation of two genomic datasets differs from the traditional formulation of the Hamming distance and we describe the computation used in determining the distance later in this section.

Before we proceed with a detailed description of the tasks, we would like to note that the specification of the tasks, including all information about the participants' datasets that should be treated as public (such as the number records in one's dataset), was provided by the competition organizers. The goal was thus to provide a secure evaluation of the specified functionality using at least the semi-honest security model (see below for detail), and the extent of the information about the other participant's data that can be deduced from the output is beyond the scope of this work.

We next describe the computation involved in the first task in more detail. The input comes as a list of SNPs, where for each SNP a number of genotypes corresponding to the individuals from the case and control groups are given. Let *P *denote the number of SNPs in the input and *N_c _*(*N_t_*) denote the number of individuals in the case (resp., control) group, whose genomic data is provided for each SNP. For each SNP, a genotype corresponding to an individual consists of two nucleotides with three possible variations denoted by AA, AB, and BB, where A and B each represent a character from the set {*A*, *T*, *C*, *G*} and are alleles in our context.

For the purpose of MAF computation, there is no distinction between case and control groups, and all individuals in both groups are treated in the same way. We denote the total number of individuals by *N *= *N_c _*+ *N_t_*. To determine MAF for a given SNP, one first needs to count the number of occurrences of alleles A and B as *n_A _*= 2*n_AA _*+ *n_AB _*and *n_B _*= 2*n_BB _*+ *n_AB_*, respectively, where *n_AA_*, *n_AB_*, and *n_BB _*denote the number of individuals with genotypes AA, AB, and BB for the given SNP. For an allele A or B, we compute its frequency as *n_A_/*2*N *or *n_B_/*2*N *, respectively, where 2 is the length of each genotype. To simplify notation, we also let *N^' ^*= 2*N *(and $N_{c}^{\prime }=2N_{c}$, $N_{t}^{\prime }=2N_{t}$). The smaller frequency corresponds to the minor allele and constitutes the output of MAF computation. We obtain the following:

**Definition 1 ***Minor allele frequency (MAF) refers to the frequency at which the least common allele occurs in a given population and is computed as*

(1) $$\begin{array}{ccc} MAF & = & \text{min}\left(n_{A},n_{B}\right)/N^{\prime }\\ & = & \text{min}\left(2n_{AA}+n_{AB},2n_{BB}+n_{AB}\right)/N^{\prime } \end{array}$$

We can simplify the computation by directly obtaining *n_A _*and *n_B _*after counting the number of times each of the two nucleotide values appears in the provided genotypes. In the case when the individuals are partitioned into two (case and control) groups, we will have *n_A _*= *n_cA _*+ *n_tA _*and *n_B _*= *n_cB _*+ *n_tB _*, where *n_cA _*and *n_tA _*represent the value of *n_A _*in the case and control groups, respectively, and similarly *n_cB _*and *n_tB _*represent the value of *n_B _*in the case and control groups. Furthermore, in our case the data are partitioned among two different entities and thus each of *n_A _*and *n_B _*need to be computed as the sum of the corresponding values at the respective sites. If we let superscripts (1) and (2) represent the values present in the genotypes of the individuals at sites 1 and 2, respectively, we now obtain and $n_{B}=n_{cB}^{\left(1\right)}+n_{tB}^{\left(1\right)}+n_{cB}^{\left(2\right)}+n_{tB}^{\left(2\right)}$. Also, now *N *corresponds to the total number of individuals in the data at both sites and in both case and control groups. Using notation similar to the above, we let $N_{c}=N_{c}^{\left(1\right)}+N_{c}^{\left(2\right)}$ and $N_{t}=N_{t}^{\left(1\right)}+N_{t}^{\left(2\right)}$.

The chi-squared test is also performed for each SNP independently, but now the data of the individuals in the case and control groups play different roles.

**Definition 2 ***Chi-squared (χ*^2^*) test is a statistical test for comparing observed data with those expected according to a specific hypothesis and is represented as *$\chi^{2}=\sum_{i=1}^{m}\frac{{\left(obs_{i}-exp_{i}\right)}^{2}}{exp_{i}}$*for some setting-dependent m*.

In our case (for a single dataset), *m *= 4 and the observed values *obs_i_*'s correspond to the observed allele counts for a SNP, namely, *n_cA_*, *n_cB_*, *n_tA_*, *n_tB_*. The corresponding expected allele counts expi's are $\left(n_{cA}+n_{tA}\right)N_{c}^{\prime }\text{/}N^{\prime }$, $\left(n_{CB}+n_{tB}\right)N_{c}^{\prime }\text{/}N^{\prime }$, $\left(n_{cA}+n_{tA}\right)N_{c}^{\prime }/N^{\prime }$, $\left(n_{cB}+n_{tB}\right)N_{t}^{\prime }/N^{\prime }$. The resulting computation can be simplified to become

(2) $$\chi^{2}=\frac{{\left(n_{cA}n_{tB}-n_{cB}n_{tA}\right)}^{2}N^{\prime }}{{N^{\prime }}_{c}{N^{\prime }}_{t}\left(n_{cA}+n_{tA}\right)\left(n_{cB}+n_{tB}\right)}$$

When the dataset is horizontally partitioned among two sites, the counts *n_cA_*, *n_tA_*, *n_cB _*, *n_tB_*, *N_c_*, *N_t_*, *N *become the sum of their respective values at both sites.

We can now proceed with the description of the computation involved in the second task, namely, the Hamming distance of two genomic datasets. In the traditional formulation of the Hamming distance, on input of two sequences of equal length, the distance is defined as the number of positions at which the corresponding symbols in the input sequences are different. This is not directly applicable to genomic sequences because they are not represented as perfectly aligned strings of the same length and thus the computation is more complex. Before we proceed with the details of the computation, we need to specify how the input (i.e., genomic datasets) are represented.

Genomic sequences are represented in the Variant Call Format (VCF), where each genomic sequence is a set of records. In each record, chromosome CHROM represents an identifier from the reference genome and position POS represents the reference position within the reference sequence CHROM. In other words, the pair ⟨CHROM, POS⟩ represents the location of the data associated with this record in the genome. The fields REF and ALT represent the reference and alternate bases, respectively, expressed as a sequence of one or more nucleotides. The field SVTYPE represents the type of the record, which is one of SUB, SNP, DEL, or INS. Only records of type SUB and SNP are used in the computation of the Hamming distance. In records of type SNP, both REF and ALT fields are one character long, while in records of type SUB, both fields can be longer. We also found that in two different inputs records at the same location ⟨CHROM, POS⟩ may be represented using different types (SUB and SNP).

**Algorithm 1 **HD(*S*_1_, *S*_2_)

1: create an empty map *M *with keys as pair ⟨CHROM, POS⟩

2: dist = 0

3: **for **each record R in *S*_1 _**do**

4:   **if **(R.SVTYPE = SUB or R.SVTYPE = SNP) **then**

5:      *M*.put(⟨R.CHROM, R.POS⟩, R)

6:      dist = dist + 1

7:   **end if**

8: **end for**

9: **for **each record R in *S*_2 _**do**

10:   **if **(R.SVTYPE = SUB or R.SVTYPE = SNP) **then**

11:      **if **(*M*.containsKey(⟨R.CHROM, R.POS⟩) = false) **then**

12:         dist = dist + 1

13:      **else**

14:         dist = dist − 1

15:         **if **(*M*.get(⟨R.CHROM, R.POS⟩).REF = R.REF and *M*.get(⟨R.CHROM, R.POS⟩).ALT ≠ R.ALT) **then**

16:            dist = dist + 1

17:         **end if**

18:      **end if**

19:   **end if**

20: **end for**

21: **return **dist

To compute the Hamming distance between two genomic datasets, we initially set the distance to 0. Then for all records in the datasets with type SUB or SNP, if a location ⟨CHROM, POS⟩ is found only in one of the datasets (and is absent in the other), the Hamming distance is incremented by 1. Also, if the location is found in both datasets and the corresponding values of the REF fields are the same, but the values of the ALT fields are different, the Hamming distance is also incremented by 1. A more detailed specification of how this procedure may be implemented is given in Algorithm 1. The algorithm uses a map to store all records of type SUB and SNP from the first dataset and (optimistically) increments the distance by 1 for each record placed in the map (lines 3-7). Then for each record of type SUB or SNP from the second dataset, if there was no record with the same location ⟨CHROM, POS⟩ in the first dataset, the distance is incremented by 1 (lines 10-12). If, however, the location is present in both datasets, the distance is first decremented by 1 (line 14). The algorithm then compares the fields REF and ALT of the records from the two datasets with the same location. If the former are equal and the latter differ, the distance is incremented by 1 (lines 15-17).

Additional information about the tasks, including examples that illustrate the computation, can be found on the competition web site [3] as well as in an article [4] being prepared by the competition organizers.

### Secure multi-party computation background

Secure multi-party computation allows two or more participants to jointly evaluate a function on their private inputs without revealing any information about the private data other than the output of the agreed-upon function. There are two standard security models used for secure function evaluation on private data that differ with respect to the types of adversaries they can tolerate. The first security model known as semi-honest (or honest-but-curious or passive) requires that all computation participants follow the computation as prescribed, but might save any information observed throughout the computation and compute with it with the goal to discover additional information about private input values. A protocol is said to be secure if no coalition of semi-honest participants (adversaries) can learn any additional information about private inputs of other parties other than what they can already compute from their legitimate output. It also follows from the security properties that any outside party is unable to learn any information about the participants' data and protocol output corresponds to evaluating the correct function on the provided data. Security in presence of semi-honest participants was a minimum security requirement for this competition.

The second, stronger, security model permits malicious (also known as active) participants who can arbitrarily deviate from the prescribed computation (and coordinate their actions). Security in this model holds if the same data protection and output correctness properties are achieved as before. Known techniques for achieving security in this model typically involve substantially larger overhead than in the semi-honest model, and we do not use it in our implementation.

There are a variety of available techniques on which secure multi-party computation protocols can be based. For the purposes of this competition, we utilize an (*n, t*)-threshold linear secret sharing scheme for representation of and secure computation over private values. With such a scheme, each private value is split into *n *secret shares (using *n *computational parties each of whom receives a share), such that combining *t *or fewer shares information-theoretically reveals no information about the private value, but combining *t *+ 1 or more shares allows the value to be reconstructed exactly. All computation proceeds on secret shares, which means that before the computation commences each participant distributes her private data among *n *computational parties and at the end of the computation reconstructs the result from the shares obtained from at least *t*+1 computation participants. We utilize Shamir's secret sharing scheme [5] and a typical way of conducting computation using this scheme requires that *t *<*n*/2. Thus we use *n *= 3 computational parties and set *t *= 1 (i.e., the parties are assumed not to collude).

In both tasks of the challenge, there are two parties who contribute their input. They will play the role of computational parties together with another party who contributes no input. As mentioned above, each input provider produces secret shares of her data and distributes them among the participants and reconstructs the output at the end of the computation. We assume that the three computational parties are connected by pair-wise secure authenticated channels (that provide secrecy and authenticity) with each other, which can be achieved using standard means.

The underlying secret sharing scheme [5] requires that shares are represented as elements of a field, which means that the input needs to be provided in the form of integer values. With a linear secret sharing scheme, a linear combination of secret-shared values can be performed by each computational party locally, without any interaction. Multiplication of two secret-shared values, on the other hand, requires communication between all of them and is treated as an elementary building block of secure protocols (we assume the multiplication protocol from [6]). These operations are typically used as the main building blocks in more complex computations, e.g., comparisons and divisions.

We utilize a number of efficient protocols for integer computation that have previously been shown secure in the standard security model. It is also known that by invoking the composition theorem [7], secure building blocks can be combined together to achieve security of the overall computation. The building blocks that will be used in the computation of the two tasks are listed next (as mentioned before, all correspond to integer computation). When performance of a building block depends on the size of the arguments provided into the function, the size is listed as a separate argument.

• [*z*] ← Mult([*x*], [*y*]) is a multiplication protocol that on input two secret-shared values *x *and *y *outputs a secret-shared product *z *= *xy*.

• [*b*] ← EQ([*x*], [*y*], *ℓ *) is an equality protocol that on input two secret-shared values *x *and *y *of bitlength at most *ℓ *outputs a bit *b *which is set to 1 iff *x *= *y*.

• [*b*] ← LT([*x*], [*y*]*, ℓ *) is a comparison protocol that on input two secret-shared values *x *and *y *of bitlength at most *ℓ *outputs a bit *b *which is set to 1 iff *x *<*y*.

• [*z*] ← Div([*x*], [*y*]*, f*) is a division protocol that on input two secret-shared values *x *and *y *of bitlength at most *f *outputs a secret-shared quotient *z *that satisfies *z *= ⌊ *x*/*y*⌋.

As shown above, each protocol takes shares of its input and produces shares of the output. It means that these protocols can be naturally and securely invoked as part of larger computation and we use them as steps in larger computation. In our implementation, we use Mult from [6], EQ and LT from [8], and Div from [9], and we refer the reader for the details of these protocols to the respective publications.

Performance of secure computation protocols is of a paramount importance for their practical use. In the case of techniques based on secret sharing, the computation is normally lightweight and thus performance is measured in terms of two parameters: (i) the number of interactive operations (e.g., multiplications) necessary to perform the computation and (ii) the number of sequential interactions, i.e., rounds. Our goal is to minimize both of these parameters for the computation performed for each task.

Before we conclude this section, we would like to say that other options for securely evaluating the functions of the competition tasks are possible. In particular, the garbled circuit evaluation approach [10] allows any function to be securely evaluated in the two-party setting. Similarly, any function can be evaluated using homomorphic encryption, or special-purpose building blocks such as private set intersection (e.g., [11]) can be used as the basis for building a custom solution for a task of the competition. Furthermore, secure computation compilers such as Fairplay [12], Sharemind [13], PICCO [14], etc. are able to produce secure implementations given function specification in a form of a program. This competition, however, allowed for custom solutions that can tune general building blocks to the needs of the tasks and result in improved performance. Because no secure implementations of the competition tasks were available to us prior to the competition, we are unable to directly compare performance of different approaches in this paper.

## Methods

### Secure distributed GWAS computation

In this section we describe our approach to securely computing the task of distributed GWAS computation, namely, computing minor allele frequencies and chi-squared statistics.

According to the task specification, the size of the input at each site, i.e., the number of SNPs and the number of individuals in the case and control groups, are treated as public and are not protected. This means that parameters *P*, $N_{c}^{\left(1\right)}$, $N_{c}^{\left(2\right)}$, $N_{t}^{\left(1\right)}$ and $N_{t}^{\left(2\right)}$ are known to all computation participants. All remaining data (i.e., the genotypes themselves) are private.

In what follows, we first describe a basic version of our solution and then provide optimization techniques that improve the runtime of program execution.

#### Basic solution

For each SNP in the input, the computation is identical (and independent of other SNPs) and thus it suffices to describe the computation for a single SNP.

We divide the overall computation into three phases: input preparation, computation execution, and output reconstruction, which proceed as follows. Observe that each input site *i *can locally compute $n_{cA}^{\left(i\right)}$, $n_{tA}^{\left(i\right)}$, $n_{cB}^{\left(i\right)}$, $n_{tB}^{\left(i\right)}$ for each SNP. This is what is done as part of input preparation, after which each input site secret shares each of its computed values and distributes the shares among all three computational parties. We use notation [*a*] to denote that the value of *a *is secret-shared among the computational parties (i.e., each party holds a different share of *a*).

During computation execution, the computation proceeds on the shares to compute MAF and chi-squared statistics using equations 1 and 2 and secure building blocks from the previous section. We choose to perform only the private portion of the computation on secret shares, while postponing the computation with public constants to the output reconstruction phase. This is done for performance reasons to reduce the size of values used in the computation.

To calculate the MAF for each SNP in parallel, the computation follows equation 1 with provisions to make the computation data-oblivious. That is, each computational party performs the following steps: In this section we describe our approach to securely

1 $\left[n_{A}\right]=\left[n_{cA}^{\left(1\right)}\right]+\left[n_{tA}^{\left(1\right)}\right]+\left[n_{cA}^{\left(2\right)}\right]+\left[n_{tA}^{\left(2\right)}\right];$

2 $\left[n_{B}\right]=\left[n_{cB}^{\left(1\right)}\right]+\left[n_{tB}^{\left(1\right)}\right]+\left[n_{cB}^{\left(2\right)}\right]+\left[n_{tB}^{\left(2\right)}\right];$

3 $\left[b\right]=\text{LT(}\left[n_{A}\right],\left[n_{B}\right],\ell_{1}\text{);}$

4 $\left[res_{1}\right]=\text{Mult}\left(\left[b\right],\left[n_{A}\right]-\left[n_{B}\right]\right)+\left[n_{B}\right];$

The first two steps that aggregate the input values are local to each computational party, but steps 3 and 4 that produce the minimum of *n_A _*and *n_B _*involve joint computation by all of them. We subsequently discuss the choice of the parameter *ℓ *_1_.

To compute the chi-squared statistics for each SNP in parallel, we similarly follow the computation in equation 2 using the following steps:

1 $\left[n_{cA}\right]=\left[n_{cA}^{\left(1\right)}\right]+\left[n_{cA}^{\left(2\right)}\right];$

2 $\left[n_{tA}\right]=\left[n_{tA}^{\left(1\right)}\right]+\left[n_{tA}^{\left(2\right)}\right];$

3 $\left[n_{cB}\right]=\left[n_{cB}^{\left(1\right)}\right]+\left[n_{cB}^{\left(2\right)}\right];$

4 $\left[n_{tB}\right]=\left[n_{tB}^{\left(1\right)}\right]+\left[n_{tB}^{\left(2\right)}\right];$

5 [*a*] = Mul([*n_cA_*], [*n_tB_*]);

6 [*b*] = Mul([*n_cB_*], [*n_tA_*]);

7 [*c*] = Mul([*n_cA_*] + [*n_tA_*], [*n_cB_*] + [*n_tB_*]);

8 [*d*] = Mul([*a*] *− *[*b*], [*a*] *− *[*b*]);

9 [*res*_2_] = Div(*k *· [*d*], [*c*]*, ℓ *_2_);

Lines 5, 6, and 8 compute the numerator in equation 2 and line 7 its denominator (multiplication by public *N *, *N_c_*, and *N_t _*is omitted). The numerator is then scaled up by a factor of *k *to ensure that using integer division will provide sufficient precision of the result. The bitlength of *k *will be on the order of the precision of the answer in bits. We defer discussion of the choice of *ℓ *_2 _to the next section.

At the end of the computation, all computational parties send their shares of the result *res*_1 _and *res*_2 _for each SNP to one of the input sites who reconstruct the values. The output party then sets the result of MAF computation to *res*_1_/*N*^*' *^and the result of the chi-squared computation to $\left(res_{2}\cdot N^{\prime }\right)/\left(kN_{c}^{\prime }N_{t}^{\prime }\right)$.

#### Optimizations

We applied several optimizations to the computation to improve its runtime.

1 The nature of the computation in this task allows all interactive operations to run in parallel in a single batch for all SNPs. That is, all *P *comparisons corresponding to line 3 of MAF computation are executed simultaneously. The same applies to line 4 of MAF computation and lines 5-9 of chisquared computation.

We can further reduce the number of rounds in chi-squared computation by running interactive independent operations at the same time. In particular, this means that lines 5-7 of the computation can be executed in a single round.

2 We modify chi-squared computation to use floating point instead of integer division after converting both operands *d *and *c *to floating point representation. This is primarily driven by the fact that performance of division we rely on (described in [15,9]) depends on the maximum of the bitlengths of its arguments and we can use substantially shorter values with floating point division compared to integer division (i.e., the bitlength can be comparable to that of *k *instead of the sum of the bitlengths of *d *and *k*). The savings noticeably outweigh the cost of integer-to-floating point conversion, or normalization (to use floating point division we need to normalize two values, while integer division needs to compute normalization of one of its arguments). We additionally slightly optimize integer to floating point conversion and floating point division compared to those given in [9] using information known about *d *and *c *(e.g., the fact that they are positive).

3 For performance reasons, we want to set parameters *ℓ *_1 _and *ℓ *_2 _(as well as the bitlength of secret shared values) to their minimum values that guarantee correctness. When the bitlength of the arguments to both comparison and division differ, the larger value is to be used. In particular, for *ℓ *_1_, the largest value of *n_A _*or *n_B _*in the LT protocol appears when only one nucleotide is present in all genotypes in both case and control groups (i.e., max(*n_A_, n_B_*) = *N*^*' *^and min(*n_A_, n_B_*) = 0), and we set ℓ_1 _= ⌈log *N^'^*⌉ (where the extra 1 is due to the specifics of the LT operation). For *ℓ *_2_, the largest value of *d *or *c *appears when *n_cB _*= *n_tA _*= 0, which leads to $n_{cA}=N_{c}^{\prime }$, $n_{tB}=N_{t}^{\prime }$, and $d={\left(N_{c}^{\prime }\right)}^{2}{\left(N_{t}^{\prime }\right)}^{2}$, and we set $n_{tB}=N_{t}^{\prime }$, and $d={\left(N_{c}^{\prime }\right)}^{2}{\left(N_{t}^{\prime }\right)}^{2}$, and we set $\ell_{2}=\left\lceil 2\left(\text{log}{N^{\prime }}_{c}+\text{log}{N^{\prime }}_{t}\right)\right\rceil$. For integer division, this value of *ℓ *_2 _needs to be additionally incremented by the bitlength of precision *k*, but fortunately after we switch to floating point representation, we can reduce the bitlength to the desired precision of the result because the values are represented in a normalized form.

### Secure distributed genomic Hamming distance computation

We next concentrate on the second task of securely computing the Hamming distance between a pair of genomic datasets in a distributed setting.

According to the task specification, the number of records in each of the two datasets are known to all parties and we denote them as *N*_1 _and *N*_2_, respectively. The content of the records, however, is private (in particular, the values that fields CHROM, POS, REF, ALT, and SVTYPE take). Because only records with SVTYPE equal to SUB and SNP are relevant for the computation, for ease of notation we refer to them as SUB and SNP records, respectively.

The high-level idea behind our solution is as follows: we first let each input site extract SUB and SNP records from its dataset and pad the resulting set with dummy records to hide its size. After each input site secret shares its records across all computational parties, the parties then run a set operation to identify all records that appear in both dataset (conceptually similar to set intersection) using ⟨CHROM, POS⟩ as the key as well as all records that appear only in one dataset (conceptually similar to symmetric difference). We accomplish this by obliviously sorting all records from both datasets using Batcher's mergesort [2] and scanning the sorted set examining every two adjacent elements in it to determine if the Hamming distance needs to be incremented by one for that pair.

At the time of competition preparation, Batcher's mergesort was available to us as one of the best options for oblivious sorting (based on the overall amount work as well as its round complexity). It is particularly well suited to this task because it is a recursive algorithm that works by first sorting the first and the second half of its input set and then merging the sorted halves. In our setup this means that the input datasets can be pre-sorted by each input site locally and only the merge step needs to be run jointly. Unfortunately, Batcher's mergesort (including the merge step) has the drawback that the number of elements in the input set has to be a power of 2, which may unnecessarily increase the runtime.

In what follows, we start by describing in detail a basic solution in the first subsection and then discuss two optimizations in the two consecutive subsections.

#### Basic solution

As before, we divide the overall computation into three phases: input preparation, joint computation execution, and output reconstruction.

*Input preparation*. Each input site *i *extracts all SUB and SNP records from its dataset and pads them with dummy records to size *N_i _*+ 1 (we require at least one dummy record). (If the combined fraction of SUB and SNP records is guaranteed to be within a certain fraction *α *< 1 of the total size for typical genomic datasets, then the datasets can be padded to *αN_i _*+ 1 records. For this competition, *α *could not be lower than 1.) Furthermore, to meet Batcher's mergesort requirements, the input parties additionally pad the sets with dummy records so that the combined size of the two datasets is 2*^q^*, where *q *= ⌈log2(*N*1 + *N*2 + 2)⌉. We use this newly formed dataset as the input into the computation and refer to it as a "dataset".

Next, the values in each record need to be converted to integers, which we accomplish as follows:

1 The location ⟨CHROM, POS⟩ is represented as *V*_1 _= CHROM *· L *+ POS, where *L *is the maximum length of any existing human chromosome. CHROM ranges from 1 to 24 (22 autosomes, plus × and Y), and for dummy records we set *V*1 = 25*L *+ 1 to avoid overlap with real records.

2 REF and ALT fields are represented as strings of nucleotides in the input. To produce their numeric counterparts, we map each nucleotide value to a two-bit integer (e.g., *A *= 0, *C *= 1, *G *= 2, and *T *= 3) and concatenate two-bit integers from a string to form a single number. To hide information about the size of the fields, the values need to be represented using the same bitlength for all records based on the maximum string length *M*. Because shorter strings need to be padded to the maximum size, we need to ensure that strings of different sizes will always be different (i.e., the padding character cannot be one of 0-3).

Instead of introducing a separate padding character, which increases the bitlength of one character from 2 to 3 bits, we append the string length in bits at the end of the string and use 0 for padding. Thus, all strings are represented using 2*M *+log *M *bits. Let *V*_2 _and *V*_3 _denote numeric values of REF and ALT fields in a record. *V*_2 _and *V*_3 _are set to 0 for dummy records.

In our implementation with *M *= 100, we partition representation of *V*_2 _and *V*_3 _into three blocks of size (2*M *+ log *M *)/3 each. This still requires comparing all 2*M *+ log *M *bits when two such values need to be compared, but reduces the size of secret shared values and thus the cost of the corresponding arithmetic. When *M *is large, *V*_2 _and *V*_3 _can instead be set to the hash of REF and ALT strings. This would guarantee constant size representation regardless of the value of *M*.

After computing a 3-tuple (*V*_1_, *V*_2_, *V*_3_) for each record in its dataset, an input site *i *sorts the records by the *V*_1 _field to form set *S_i_*, generates shares of all records in *S_i_*, and distributes them to the computational parties (we slightly abuse notation and use [*S_i_*] to denote shares of all values in *S_i_*). It also distributes shares of the number of dummy records *d_i _*in *S_i_*.

*Computation execution*. After receiving two sorted sets of ([*V*_1_], [*V*_2_], [*V*_3_]) triples from both input sites, the computational parties run oblivious merge using [*V*1] as the key. The algorithm is built using an input-independent sequence of compare-and-exchange operations. Each operation takes two integers and either swaps them or leaves them unchanged so that the first output (*min*) is always smaller than the second (*max*). In our framework, it is implemented as follows:

1 [*c*] = LT([*a*], [*b*]*, ℓ *);

2 [*min*] = [*c*]([*a*] *− *[*b*]) + [*b*];

3 [*max*] = [*c*]([*b*] *− *[*a*]) + [*a*];

Note that lines 2 and 3 involve only a single multiplication (i.e., first compute [*c*]([*a*] *− *[*b*]) and then set [*min*] and [*max*] with no additional interaction). When applying this operation to our setting, comparisons on line 1 are performed using [*V*_1_]'s, but the entire records ([*V*_1_], [*V*_2_], [*V*_3_]) are swapped (or left unchanged) using comparison results [*c*].

The computational parties next compute the Hamming distance as specified in Algorithm 2. Sets *S*_1 _and *S*_2 _represent sorted input triples of the input parties and parameters *ℓ*_1 _and *ℓ*_2 _correspond to the bitlengths of fields *V*_1 _and *V*_2 _(or *V*_3_), as discussed previously.

Because a specific location *V*_1 _appears only once in each of the input datasets (except for dummy records), there will be at most two records with the same *V*_1 _in the combined set. The algorithm works by looking at each pair of two consecutive elements in the combined sorted set and adds 1 to dist if this is the first time the location appears on the list (i.e., *a_i _*= 0 on line 4). The distance is incremented automatically for the first record (dist = 1 on line 2). Then, if a location appears for the second time (*a_i _*= 1 on line 4), the algorithm examines the values of *V*_2 _and *V*_3 _fields (lines 5-6) to determine whether the condition for incrementing the distance is satisfied (i.e., *b_i _*= 1 and *c_i _*= 0). If not (*b_i _*= 0 or *c_i _*= 1), dist is decremented by 1 to compensate for the fact that it was increased during previous loop iteration. All dummy records collectively contribute distance −*d*_1_−*d*_2 _+2 (i.e., 0 for the first two records and −1 for each additional record) and this is why we adjust the computed distance at the end (line 9). We note that all loop iterations and all comparisons within a loop iteration can be carried out in parallel.

**Algorithm 2 **SecureHD([*S*_1_], [*S*_2_], [*d*_1_], [*d*_2_])

1: ${\left(\left[V_{1}^{\left(i\right)}\right],\left[V_{2}^{\left(i\right)}\right],\left[V_{3}^{\left(i\right)}\right]\right)}_{i=1}^{2^{q}}=\text{Merge}\left(\left[S_{1}\right],\left[S_{2}\right]\right)$

2: dist = 1

3: **for ***i *= 2 to 2*^q ^***do**

4:   $\left[a_{i}\right]=\text{EQ}\left(\left[V_{1}^{\left(i-1\right)}\right],\left[V_{1}^{\left(i\right)}\right],\ell_{1}\right)$

5:   $\left[b_{i}\right]=\text{EQ}\left(\left[V_{2}^{\left(i-1\right)}\right],\left[V_{2}^{\left(i\right)}\right],\ell_{2}\right)$

6:   $\left[c_{i}\right]=\text{EQ}\left(\left[V_{3}^{\left(i-1\right)}\right],\left[V_{3}^{\left(i\right)}\right],\ell_{2}\right)$

7: dist = dist + (1 *− *[*a_i_*]) + [*a_i_*]([*b_i_*](1 *− *[*c_i_*]) *− *1)

8: **end for**

9: dist = dist + [*d*_1_] + [*d*_2_] −2

10: **return **dist

*Output reconstruction *is straightforward and consists only of receiving and combining shares of the computed Hamming distance.

#### Separating SUB and SNP records

As the first significant optimization, we separate computation of the distance for SNP and SUB records and consequently reconstruct the overall distance from the two values. The main reason for this is to reduce the time comparisons of *V*_2 _and *V*_3 _take. Recall that SNP records contain a single character in REF and ALT fields, while SUB records can contain longer strings. In the genomic datasets we worked with, a great majority of all records were SNP records that can be processed using 2-bit comparisons for *V*_2 _and *V*_3 _(i.e., ℓ_2 _= 2). In the basic solution, however, the bitlength had to be unnecessarily increased by two orders of magnitude for most records to meet privacy requirements. Thus, the idea consists of extracting two sets from each input dataset: one consisting of SNP records and another consisting of SUB records. Then the distance for SUB records is computed separately from the distance for SNP records and the sum is returned as the result.

This strategy works well if all records with the same ⟨CHROM, POS⟩ pair are always marked with the same type across all datasets. It is, however, possible for two datasets to contain SUB and SNP records corresponding to the same location. Because of the existence of such records, the Hamming distance will not be computed correctly if we simply add the two distances together. That is, if one record appears in the SUB set and another with the same location appears in the SNP set, they collectively will contribute 2 to the overall distance instead of correct 0 or 1 (depending on other attributes). To address this, we need to find all such pairs and compensate for the difference they introduced, which is the most subtle part of our solution. We next provide more detail about the solution and highlight the differences from the basic scheme.

*Input preparation*. Given a dataset, an input entity produces two subsets: one composed of SUB records and one composed of both SUB and SNP records from the dataset. As before, both sets need to be padded with dummy records to hide their number and make the size to be a power of 2 to the combined size of 2*^qs ^*and 2*^q^*, where *q_s _*= ⌈log(*α_s_*(*N*_1 _+ *N*_2_) + 2)⌉ and *q *= ⌈log(*α*(*N*_1 _+ *N*_2_) + 2⌉ and *α_s _*(*α*) denotes the maximum fraction of SUB (resp., SNP and SUB) records in genomic datasets (we were given *α *= 1 and *α_s _*= 0.3). All records in the SUB set are converted to (*V*_1_, *V*_2_, *V*_3_) triples as before. For the SNP&SUB set, one-character REF and ALT fields in SNP or SUB records are represented using integers 0-3, while these fields of longer length in SUB records are represented using integer 4 (i.e., *V*_2 _and *V*_3 _fields are 3 bits long). This will guarantee that comparison of a one-character long REF or ALT field in a SNP record with a longer REF or ALT field in a SUB record will result in their inequality. We also add another binary attribute *V*_4 _to each record of the SNP&SUB set that indicates whether the record is of SUB type (*V*_4 _= 0) or SNP type (*V*_4 _= 1). We set *V*_4 _= 0 in dummy records.

Each input entity now produces shares of (*V*_1_, *V*_2_, *V*_3_) in its SUB set and (*V*_1_, *V*_2_, *V*_3_, *V*_4_) in its SNP&SUB set (together with the number of dummy records in each set) and distributes them to the computational parties. We note that computation with SNP&SUB sets can be performed on shorter bitlengths, which results in faster arithmetic, and thus we setup two different instances of the secret sharing scheme and process SUB sets separately from SNP&SUB sets.

*Computation execution*. To compute the Hamming distance correctly, we now distinguish between different cases: (i) SUB records that don't have a SNP record with identical location in the other dataset, (ii) SNP records that don't have a SUB record with identical location in the other dataset, and (iii) records that have another record with identical location but different type present in the other dataset. Let *N*0 denote the number of records in the third category.

**Algorithm 3 **SecureHD2([*S*_1_], [*S*_2_])

1: ${\left(\left[V_{1}^{\left(i\right)}\right],\left[V_{2}^{\left(i\right)}\right],\left[V_{3}^{\left(i\right)}\right],\left[V_{4}^{\left(i\right)}\right]\right)}_{i=1}^{2^{q}}=\text{Merge}\left(\left[S_{1}\right],\left[S_{2}\right]\right)$

2: $\text{dist}=\left[V_{4}^{\left(1\right)}\right]$

3: **for ***i *= 2 to 2*^q ^***do**

4:   $\left[a_{i}\right]=\text{EQ}\left(\left[V_{1}^{\left(i-1\right)}\right],\left[V_{1}^{\left(i\right)}\right]\right)$

5:   $\left[b_{i}\right]=\text{EQ}\left(\left[V_{2}^{\left(i-1\right)}\right],\left[V_{2}^{\left(i\right)}\right]\right)$

6:   $\left[c_{i}\right]=\text{EQ}\left(\left[V_{3}^{\left(i-1\right)}\right],\left[V_{3}^{\left(i\right)}\right]\right)$

7:   $\left[d_{i}\right]=\text{OR}\left(\left[V_{4}^{\left(i-1\right)}\right],\left[V_{4}^{\left(i\right)}\right]\right)$

8:   $\left[e_{i}\right]=\text{XOR}\left(\left[V_{4}^{\left(i-1\right)}\right],\left[V_{4}^{\left(i\right)}\right]\right)$

9:   $\text{dist}+=\left[d_{i}\right]\left(\left(1-\left[a_{i}\right]\right)\left[V_{4}^{\left(i\right)}\right]+\left[a_{i}\right]\left(\left[b_{i}\right]\left(1-\left[c_{i}\right]\right)-\left[V_{4}^{\left(i-1\right)}\right]\right)\right)-\left[a_{i}\right]\left[e_{i}\right]$

10: **end for**

11: **return **dist

The computational parties execute Algorithm 2 on two SUB datasets. This computes the distance corresponding to the records in category 1, but also introduces offset *N*_0_. The parties then execute Algorithm 3 on two SNP&SUB sets that computes the distance corresponding to categories 2 and 3 and additionally compensates for the offset. The output will then be the sum of the distances computed by both algorithms.

In Algorithm 3, when examining each pair of consecutive records, we only consider those that contain at least one SNP record within the pair (*d_i _*= 1 on line 9). Furthermore, similar Algorithm 2, when observing a location for the first time, we add 1 to the Hamming distance, but only if it is a SNP record ($V_{4}^{\left(1\right)}=1$ on line 2 and $V_{4}^{\left(i\right)}=1$ on line 9). If a location appears for the second time, we undo the previous increment if *b_i _*= 0 or *c_i _*= 1 as before, but only if the record preceding the current one is of type SNP (i.e., $V_{4}^{\left(i-1\right)}=1$ on line 9). By doing that, we are able compute the distance corresponding to records of second and third types without introducing errors. The offset *N*_0 _is compensated by the last term *a_i_e_i _*on line 9, that counts the number of pairs of consecutive records that have the same location (*a_i _*= 1), but different types (*e_i _*= 1). OR([*x*], [*y*]) and XOR([*x*], [*y*]) are implemented as [*x*]+[*y*]*−*Mult([*x*], [*y*]) and [*x*]+[*y*]*−*2Mult([*x*], [*y*]), respectively (computation of *d_i _*and *e_i _*reuses the same multiplication result).

Note that dummy records do not introduce any error in Algorithm 3. That is, *d_i _*= 0 and *e_i _*= 0 when both records *i *and *i − *1 are dummy and the expression on line 9 evaluates to 0. Similarly, when record *i−*1 is real while record *i *is fake that expression also evaluates to 0 because *a_i _*= 0 and $V_{4}^{\left(i\right)}=0$.

After computing the distances corresponding to SUB and SNP&SUB sets, the parties need to convert shares of one of them into shares of the same value in the secret sharing setup used by the other algorithm. Then the distances can be locally added to compute the over-all result. *Output reconstruction *is performed as before by exchanging the shares and recovering the result.

The performance gain achieved by this optimization highly depends on the values of public parameters *α_s_*, *α*, and *M*. While the gain stems from using shorter values for *V*_2 _and *V*_3 _with SNP&SUB sets, the total number of records processed using this solution $\left(2^{q}+2^{q_{s}}\right)$ is greater than in the basic scheme (2*_q_*). Therefore, this optimization is recommended with relatively small *α_s _*and large *M*. In our experiments with *α_s _*= 0.3, *α *= 1, and *M *= 100, we observed approximately 30% performance improvement compared to the basic scheme.

#### Reducing set size

Our second optimization is with respect to oblivious sort and removing the requirement that the input size has to be a power of 2 for the merge step of Batcher's mergesort. To explain how our optimization works, we need to provide additional details about Batcher's mergesort algorithm.

Recall that the merge step takes two sorted sets *L*_1 _= (*a*_1_, *a*_2_, ..., *a_m_*) and *L*_2 _= (*b*_1_, *b*_2_,..., *b_n_*), where *m *+ *n *is a power of 2. It first combines them into a single sequence that first monotonically increases and then decreases as *L *= (*a*_1_, *a*_2_, ..., *a_m_, b_n_, ..., b*_2_*, b*_1_), after which a sequence of compare-and-exchange operation is executed as specified by the following pseudo-code:

$$\begin{array}{c} \text{for }\left(r=\left(m+n\right)/2;r>0;r=r/2\right)\\ \;\text{for }\left(j=0;j<m+n;j=j+2r\right)\\ \;\;\text{for }\left(k=j;k<j+r;k=k+1\right)\\ \;\;\;\;\;\text{compare - and - exchange}\left(L\left[k\right],L\left[k+r\right]\right) \end{array}$$

After executing the first iteration of the outer loop, the first (second) half of *L *will contain (*m *+ *n*)/2 smallest (resp., largest) elements of *L *although they are not necessarily sorted. After its second iteration, the *i*th quarter of *L *will contain the *i*th quarter of elements in the final sorted list for *i *= 1, ..., 4. This process continues until each sublist contains one element and *L *becomes sorted. Notice that the algorithm uses log(*m *+ *n*) iterations of the outer loop, and in each iteration every element in the list is used in a compare-and-exchange operation, which is the reason for requiring the size of the list to be a power of 2.

Consider an example with input *L*_1 _= (3, 4, 6, 9, 12) and *L*_2 _= (2, 5, 10), which is combined into *L *= (3, 4, 6, 9, 12, 10, 5, 2). In the first iteration of the outer loop, compare-and-exchange operations are performed on pairs (3, 12), (4, 10), (6, 5), and (9, 2), and the resulting list is (3, 4, 5, 2, 12, 10, 6, 9). In the second iteration, comparisons are performed on (3, 5), (4, 2), (12, 6), (10, 9) and produce (3, 2, 5, 4, 6, 9, 12, 10). In the last iteration, we compare every pair of consecutive elements (3, 2), (5, 4), (6, 9), (12, 10), which results in the final sorted list (2, 3, 4, 5, 6, 9, 10, 12). If *L*2 = (2, 5) instead, after all iterations 2 will remain at the end of the list making it unsorted, as the element does not have any pair to use in a comparison.

We next proceed with describing our strategy for generalizing the merge operation to work with inputs of arbitrary sizes, which might be of independent interest. There will be no need to pad the input in the beginning to make the overall input size to be a power of 2, but dummy records are now added throughout algorithm execution as needed. This means that earlier loop iterations use a smaller number of elements and are therefore faster than in the original algorithm. In particular, at each loop iteration, if the size of a sublist is odd, we append a copy of its last element to the end. This will ensure that comparisons can be performed at the current level while still preserving the necessary properties of the (partially) sorted list. For example, suppose we want to merge (3, 6, 8) and (5, 7). Before the first iteration 5 will be added to the list (3, 6, 8, 7, 5) because the number of elements in it is odd, and we obtain (3, 5, 5, 7, 6, 8) at the end of that iteration. At the time of second iteration, the size of sublists (3, 5, 5) and (7, 6, 8) is also odd and they are modified to be (3, 5, 5, 5) and (7, 6, 8, 8). In the next iteration no additional padding is used and we obtain (3, 5, 5, 5, 6, 7, 8, 8) at the end of the algorithm.

In the context of Hamming distance computation, we similarly make a copy of the entire last record of a subset as needed during the merge step. More importantly, after having the list sorted, we need to ensure the Hamming distance is computed correctly because the introduction of repeated records creates inaccuracies in Algorithm 2. Now two consecutive records with the same location in the sorted set may correspond to (i) two records in the original datasets, (ii) two copied records, or (iii) one original and one copied record. Let *a_i _*and *a_j _*be two different records with the same location in the original datasets. If they get copied during the merge as $a_{i}^{\prime }$ and $a_{j}^{\prime }$, the relative order of these records in the sorted list can be arbitrary (e.g., $\left(a_{i}^{\prime },a_{i},a_{j},a_{j}^{\prime }\right)$, $\left(a_{i}^{\prime },a_{j},a_{i},a_{j}^{\prime }\right)$, etc.) and they may contribute more than 1 to the computed distance.

We address the problem by modifying locations of records in the datasets so that (i) two records originally with the same location are assigned locations that differ by 1 and (ii) two records originally with different locations are assigned locations that differ by more than 1. By doing that, a pair of consecutive records with the same location in the sorted set is guaranteed to correspond to either two copied records or one original record and its copy. In either case, the Hamming distance should not get affected. We implement this change by setting the location to 4*V*_1_, where *V*_1 _is the original location, for records from the first input site and to 4*V*_1 _+ 1 for records from the second input site.

**Algorithm 4 **SecureHD3([*S*_1_], [*S*_2_])

1: ${\left(\left[V_{1}^{\left(i\right)}\right],\left[V_{2}^{\left(i\right)}\right],\left[V_{3}^{\left(i\right)}\right]\right)}_{i=1}^{2^{q}}=\text{NewMerge}\left(\left[S_{1}\right],\left[S_{2}\right]\right)$

2: dist = 1

3: **for ***i *= 2 to 2*^q ^***do**

4:   $\left[a_{i}\right]=\text{EQ}\left(\left[V_{1}^{\left(i-1\right)}\right],\left[V_{1}^{\left(i\right)}\right]\right)$

5:   $\left[b_{i}\right]=\text{EQ}\left(\left[V_{2}^{\left(i-1\right)}\right],\left[V_{2}^{\left(i\right)}\right]\right)$

6:   $\left[c_{i}\right]=\text{EQ}\left(\left[V_{3}^{\left(i-1\right)}\right],\left[V_{3}^{\left(i\right)}\right]\right)$

7:   $\left[d_{i}\right]=\text{EQ}\left(\left[V_{1}^{\left(i-1\right)}\right]+1,\left[V_{1}^{\left(i\right)}\right]\right)$

8:   $\text{dist}+=\left(1-\left[a_{i}\right]\right)\left(1-\left[d_{i}\right]+\left[d_{i}\right]\left(\left[b_{i}\right]\left(1-\left[c_{i}\right]\right)-1\right)\right)$

9: **end for**

10: **return **dist

With this solution, the input sites prepare their input datasets as in the basic scheme, but pad a set to size *N_i _*+ 1 instead of requiring the combined size to be a power of 2. The computational parties can locally modify *V*_1_'s in the input records run the improved merge and compute the Hamming distance as specified in Algorithm 4. The algorithm has two major differences compared to Algorithm 2: (i) when examining each pair of consecutive records, only pairs with different locations can contribute to the distance (*a_i _*= 0 on line 8) and (ii) when locations of two consecutive records differ by 1 (*d_i _*= 1 on line 8), they are treated as having the same location in Algorithm 2, and when the locations differ by neither 1 nor 0 (*d_i _*= 0 and *a_i _*= 0 on line 8), they are treated as having different locations in Algorithm 2.

Dummy records inserted by each input site into their input datasets do not affect correctness of the Hamming distance that uses this optimization (including the combined solution in Algorithm 5). This is because the first dummy record from the first input set will result in the distance incremented by 1, while the first dummy record from the second input set will result in the distance decremented by 1. All consecutive dummy records from the first or the second input datasets do not modify the distance (because all records with the same *V*_1 _are ignored).

We recently became aware of a sorting algorithm [16] that generalizes Batcher's bitonic sort to input sizes which are not a power of 2 without adding extra records during the sorting procedure. The algorithm results in the same asymptotic complexity as our solution, but performs fewer compare-and-exchange operations in each iteration (because dummy records are not added), which is expected to lead to better performance than our algorithm. We plan to provide both theoretical and empirical comparison of this algorithm with our solution as future work.

Our final solution consists of using both optimizations from the previous and current subsections, and we summarize it in Algorithm 5. We omit its explanation due to space considerations.

**Algorithm 5 **SecureHD4 $\left(\left[S_{1}^{\text{SUB}}\right],\left[S_{1}^{\text{SNP}}\right],\left[S_{2}^{\text{SUB}}\right],\left[S_{2}^{\text{SNP}}\right]\right)$

1: dist_1 _= SecureHD3$\left(\left[S_{1}^{\text{SUB}}\right],\left[S_{2}^{\text{SUB}}\right]\right)$

2: ${\left(\left[V_{1}^{\left(i\right)}\right],\left[V_{2}^{\left(i\right)}\right],\left[V_{3}^{\left(i\right)}\right],\left[V_{4}^{\left(i\right)}\right]\right)}_{i=1}^{2^{q}}=\text{NewMerge}\left(\left[S_{1}^{\text{SNP}}\right],\left[S_{2}^{\text{SNP}}\right]\right)$

3: $\text{dis}{\text{t}}_{2}=\left[V_{4}^{\left(0\right)}\right]$

4: **for ***i *= 2 to 2*^q ^***do**

5:   $\left[a_{i}\right]=\text{EQ}\left(\left[V_{1}^{\left(i-1\right)}\right],\left[V_{1}^{\left(i\right)}\right]\right)$

6:   $\left[b_{i}\right]=\text{EQ}\left(\left[V_{2}^{\left(i-1\right)}\right],\left[V_{2}^{\left(i\right)}\right]\right)$

7:   $\left[c_{i}\right]=\text{EQ}\left(\left[V_{3}^{\left(i-1\right)}\right],\left[V_{3}^{\left(i\right)}\right]\right)$

8:   $\left[d_{i}\right]=\text{OR}\left(\left[V_{4}^{\left(i-1\right)}\right],\left[V_{4}^{\left(i\right)}\right]\right)$

9:   $\left[e_{i}\right]=\text{XOR}\left(\left[V_{4}^{\left(i-1\right)}\right],\left[V_{4}^{\left(i\right)}\right]\right)$

10:   $\left[g_{i}\right]=\text{EQ}\left(\left[V_{1}^{\left(i-1\right)}\right]+1,\left[V_{1}^{\left(i\right)}\right]\right)$

11:   $\text{dis}{\text{t}}_{2}+=\left(1-\left[a_{i}\right]\right)\left(\left[d_{i}\right]\left(\left(1-\left[g_{i}\right]\right)\left[V_{4}^{\left(i\right)}\right]+\left[g_{i}\right]\left(\left[b_{i}\right]\left(1-\left[c_{i}\right]\right)-V_{4}^{\left(i-1\right)}\right)\right)-\left[g_{i}\right]\left[e_{i}\right]\right)$

12: **end for**

13: **return **dist_1 _+ Convert(dist_2_)

## Results

In this section, we provide experimental results of securely computing GWAS statistics and the Hamming distance in a distributed setting. We ran experiments in LAN and WAN settings with three computational parties connected by pair-wise secure authenticated channels with each other. The LAN experiments were conducted using 2.4 GHz 6-core Red Hat Linux machines connected through 1 Gb/s Ethernet with pairwise round-trip times 0.3 msec. Our WAN experiments used two machines from the LAN setting and employed another 2.1 GHz 8-core machine from the GENI infrastructure [17] at a different geographic location with Red Hat Linux. The pairwise round-trip times between these machines were 0.3 msec, 9.2 msec, and 9.2 msec. Each GWAS experiment was run 20 times and each Hamming distance experiment was run 5 times and the median over all runs is reported.

For GWAS computation, case and control groups consisted of genotypes of 200 individuals each (100 individuals at each input site in each group). We measured the runtime of the MAF and chi-squared computation by varying the number of SNPs in the input. The results are given in Table 1. Modulus size (Mod) corresponds to the bitlength of secret shared values.

**Table 1:** Performance of GWAS computation in LAN and WAN settings (in seconds)

No. of SNPs	Mod (bits)	MAF		Chi-squared test	
		
		LAN	WAN	LAN	WAN
500	84	0.062	0.211	2.210	6.950

1000	84	0.121	0.376	4.456	12.03

2000	84	0.244	0.621	8.900	21.73

4000	84	0.496	1.200	17.98	39.59

8000	84	1.005	2.251	35.78	76.01

Our implementation incorporates all optimizations and uses parameters *ℓ *_1 _= 11 and *ℓ *_2 _= 21 computed as described in the optimizations subsection of the secure GWAS computation section (*ℓ *_2 _= 35 + *|k| *would be required for integer division, but a lower parameter is requested precision). We can see from the table that sufficient with floating point operation to obtain the the execution time is linear in the number of SNPs in both settings, and the overhead in WAN is almost three times as large as that in LAN, which is primarily due to larger communication delays in WAN. Another observation not present in the table is that division performed in chi-squared computation contributes almost the entire runtime (close to 99%) and thus any optimizations applied to division can lead to direct improvement of chi-squared performance.

For the Hamming distance computation, we conducted four sets of experiments in the LAN setting, that correspond to the basic scheme (i) with no optimizations, (ii) with the first optimization, (iii) with the second optimization, and (iv) with both optimizations. By comparing execution times of different schemes, we can see performance gains from different optimizations. In the WAN setting, we only report the timings of the best (last) scheme. For each set of experiments, we varied the number of records in the genomic dataset at each input site. The results are presented in Table 2.

**Table 2:** Performance of Hamming distance computation in LAN and WAN settings (in seconds)

Scheme	Setting	Mod (bits)	Genomic dataset size				
			
			5000	10000	20000	40000	80000
Basic	LAN	118	123.9	256.7	524.6	1086	2212

1st opt.	LAN	118, 85	89.29	187.9	395.1	823.3	1705

2nd opt.	LAN	118	114.4	232.8	472.4	962.7	1957

Both opt.	LAN	118, 85	73.15	151.4	314.4	654.6	1343

Both opt.	WAN	118, 85	154.5	314.1	614.4	1244	3367

We used two different secret sharing bitlengths for schemes that apply the first optimization (one for the computation with SUB records and another for the computation with SNP&SUB records). The complexity of the merge is *O*(*n *log *n*) for combined sequences of size *n *and computing the distance itself is linear in *n *(with larger constants), which the runtimes in Table 2 follow. In the LAN setting, the two optimizations result in performance improvement up to 27.9% and 13.1% on our set of parameters when applied separately, and 40.9% when applied together. Performance gain of the first optimization heavily depends on parameters (*α*, *α_s_*, and *M *), while the gain of the second optimization depends on the difference between the combined input size *N *and 2^⌈log *N*⌉^. The the smaller the difference is, the smaller improvement is expected.

## Conclusions

In this work we report on our experience with participation in the 2015 iDASH secure genomic computation competition. We show how to securely compute MAF and chi-squared statistics in the context of GWAS computation and the Hamming distance between two genomic datasets and report on their performance results. We develop a number of novel optimizations, some of which may be of independent interest.

## Competing interests

The authors declare that they have no competing interests.

## Authors' contributions

Marina Blanton and Yihua Zhang designed the protocols for distributed GWAS computation and secure sequence comparison in the form of Hamming distance. Yihua Zhang and Ghada Almashaqbeh implemented the protocols and reported evaluation results. Marina Blanton and Yihua Zhang drafted the manuscript for publication. Ghada Almashaqbeh contributed to the work while at the University of Notre Dame.

## Declarations

Publication funding for this supplement was supported by iDASH U54HL108460, iDASH linked R01HG007078 (Indiana University), NHGRI K99HG008175 and NLM R00LM011392.
